# Supplementary material for: Bovine schistosomiasis in some selected areas of South Wollo and Oromia Zones of Amhara Region, North-East Ethiopia
Source: PLoS One. 2022 Jun 17;17(6):e0259787. doi: 10.1371/journal.pone.0259787 (PMC9205502; doi:10.1371/journal.pone.0259787)
Supplement: S2 File — (DOC) [file pone.0259787.s004.doc]

| **Condition** | **Score** | **Lumbar region** | **Rib cage** | **Sternum** |
| --- | --- | --- | --- | --- |
| **Starving** | **0** | Extremely emaciated and on the point of death. It is not possible to detect any muscle or fatty tissue between the skin and the bone. | Skin is sunken between visible ribs. | There is no sternal fat. |
| **Very thin** | ***1*** | The spinous processes are prominent and sharp. The transverse process are also sharp, the fingers pass easily under the ends, and it is possible to feel between each process. The eye muscle areas are shallow with no fat cover. | Ribs are clearly visible. | Sternal fat is easily grasped and moved from side to side. |
| **Thin** | ***2*** | The spinous processes feel prominent but smooth, and individual processes can be felt only as fine corrugations. The transverse processes are smooth and rounded, and it is possible to pass the fingers under the ends with a little pressure. The eye muscle areas are of moderate depth, but have little fat cover. | Some ribs can be seen.  There is a small amount of fat cover.  Ribs are still felt. | Sternal fat is wider and thicker but can still be grasped and moved slightly from side to side. |
| **Moderate** | ***3*** | The spinous processes are detected only as small elevations; they are smooth and rounded and individual bones can be felt only with pressure. The transverse processes are smooth and well covered, and firm pressure is required to feel over the ends. The eye muscle areas are full, and have a moderate degree of fat cover. | Ribs are barely seen; an even layer of fat covers them. Spaces between ribs are felt using pressure. | Sternal fat is wide and thick.  It can still be grasped but has very little movement. |
| **Fat** | ***4*** | The spinous processes can just be detected with pressure as a hard line between the fat covered eye muscle areas. The ends of the transverse processes cannot be felt. The eye muscle areas are full, and have a thick covering of fat. | Ribs are not seen. | Sternal fat is difficult to grasp and cannot be moved from side to side. |
| **Very fat** | ***5*** | The spinous processes can't be detected even with firm pressure, and there is a depression between the layers of fat in the position where the spinous processes would normally be felt. The transverse processes cannot be detected. The eye muscle areas are very full with thick fat cover. There may be large deposits of fat over the rump and tail. | Ribs are not visible and are covered with excessive fat. | Sternal fat extends and covers the sternum. It cannot be grasped |

Source (https://catalog.extension.oregonstate.edu.)

NB: In this study the body condition score were categorized in to poor, medium and good. The score from 0 and 1 categorized as poor, 2 and 3 as medium and 4 and 5 as good body condition.
