# Supplementary material for: Bovine schistosomiasis in some selected areas of South Wollo and Oromia Zones of Amhara Region, North-East Ethiopia
Source: PLoS One. 2022 Jun 17;17(6):e0259787. doi: 10.1371/journal.pone.0259787 (PMC9205502; doi:10.1371/journal.pone.0259787)
Supplement: Attachment — Submitted filename: Manuscript_reviewed_track change.doc [file pone.0259787.s005.doc]

**BOVINE SCHISTOSOMIASIS IN SOME SELECTED AREAS OF SOUTH WOLLO AND OROMIA ZONES OF AMHARA REGION, NORTH-EAST ETHIOPIA**

**Author**s:

Gashaw Molla ^1^, Tarekegn Tintagu^2*^, Ahmed Yasin^2^, Bethelehem Alemu ^2^, Alula Alemayehu Assen^2^ and Kassahun Tadesse ^2^.

**Affiliation:**

1. Dessie Zuria Livestock Development Office, Dessie, Ethiopia
2. Wollo University, School of Veterinary Medicine, Dessie, Ethiopia

***Corresponding author**

E-mail: drtarekegn@yahoo.com (TT)

# ABSTRACT

*Schistosoma bovis* is among the schistosome species of veterinary importance which is responsible for considerable economic losses in livestock industry. A cross sectional study was conducted in some selected districts of South Wollo and Oromia Administrative Zones, Amhara regional state, Ethiopia from November 2020 to June 2021 to determine the prevalence of bovine schistosomiasis and to characterize pathological lesions induced by Schistosoma bovis. For coprological examination total of 768 fecal samples were collected from cattle both from the field and the abattoirs using simple random and systematic sampling techniques respectively. In addition to the coprological examination, postmortem examination was carried out at Kombolcha and Dessie municipal abattoirs on 384 cattle which came from various livestock markets from the selected districts to recover the adult worms from mesenteric veins, and to characterize the gross lesions in liver and intestinal tracts. Chi-square (χ^2^) test at 95% confidence interval was computed to estimate the association of the risk factors with the occurrence of schistomaisis and Kappa test was employed to understand level of measurement of interrater reliability among the two tests.. Of the total of 768 faecal samples examined, 128 (16.7 %) were found to be positive for Schistosoma *bovis* eggs. The prevalence of schistomaisis was statistically significantly associated with body condition (χ^2^= 28.442; P-value = 0.000); breed (χ^2^ = 10.963; P-value = 0.001) and origin (χ^2^ = 9.699; P-value =0.046) of the study animals. However; the occurrence Schistosomiasis had negative association with sex and age of the animals. Findings of the abattoir survey showed that 55(14.3%) cattle were positive for schistosoma parasite egg and 66 (17.2%) cattle were found to be positive for adult worm in their mesenteric veins, these findings showed that there was strong measure of agreement (Kappa=0.775) between coprological and post-mortem examination. In the present study schistosoma lesions were identified only in the liver and intestinal tracts. The major gross lesions observed in the liver were whitish foci scattered under the capsule and on the cut surface of the liver and fibrosis and thickening of the portal tracts, whereas in the intestinal tracts thickened and hyperaemic mucosa, with focal petechial hemorrhages, ulcerations, and superficial intestinal veins were occluded by thrombi. The findings obtained from this research work showed that bovine Schistosomiasis is major animal health problems in the study areas, therefore; intensify effort is needed to address risk factors contributing to the epidemiology and control of the disease.

**Key words:** Bovine schistosomiasis; Pathology; Prevalence; Risk factors; South Wollo and Oromia Administrative zones.

# INTRODUCTION

Ethiopia has the largest number of livestock in Africa [1-2]. Livestock contribute to over 40% of the value of annual agricultural production and no less 15% the gross national product (GNP). However, the livestock sector is not efficiently and fully exploited due to several constraints like malnutrition, traditional husbandry practice, poor genetic makeup and prevailing diseases [3-4].

Parasitic diseases have been indicated among the major constraints in animal production throughout the tropical and sub-tropical countries of the world. Trematodes have been reported as one of the major problems both in animals and humans around the world [5-7].

Schistosomiasis is one of the trematode infection It has been indicated as endemic in sub-Saharan Africa [8-9]. Water lodged and marshy grazing field, a condition anticipated for being ideal for the propagation and maintenance of the intermediate host (snails) has been found responsible for high prevalence of the infection [6]. The disease has been indicated to be responsible for considerable economic losses in livestock industry, mainly through mortality, reduced fertility, productivity and stunted growth [10-11]. Routine diagnosis of intestinal Schistosomiasis relies heavily on observation of clinical signs and faecal examination for eggs of the parasite [12]. Schistosomiasis control focuses on reducing disease through periodic, large-scale population treatment with praziquantel; a more comprehensive approach including potable water, adequate sanitation, and snail control [13].

Ethiopia is highly endemic for Schistosomiasis, since temperature in Ethiopia appears to be the major factor that affects the distribution of Schistosoma species [14]. Out of 10 species reported to naturally infected cattle, six have received particular attention mainly because of their recognized veterinary significance. The major species that cause animal schistosomiasis include: Schistosoma bovis, S. indicum, S. japonicum, S. matthei, S. intercalatum,  S. nasale and S. rodhoni . S. bovis, S. matthei and  S. intercalatum are the most important *s*pecies that can cause Schistosomiasis in ruminants [7].

The geographical distribution of bovine schistosomiasis has been determined primarily by the distribution of snail intermediate host particularly Bulinus species which are important for the occurrence of disease in bovine species. Schistosoma bovis is a species whose final hosts are bovines, ovines, caprines and whose secondary hosts are small wild ruminants.

Schistosoma bovis is the main cause of cattle schistosomiasis. It has localized distribution, which is found commonly in northern, eastern, southwestern and central parts of Ethiopia [15]. Reports on animal schistosomes are very scanty and epidemiological studies conducted on bovine schistosomiasis are suggestive of the endemicity of the disease particularly in the area with large permanent water bodies and marsh pasture area [16].

The prevalence of *Schistosoma bovis* has been reported from different regions of the country by a number of authors; 28% in Kemissie by Ameni et al. [17] in Fogera it was 10.17% by Mengistu et al. [18], and 13.70% by Mersha *et al.* [16], in Debre Tabor it was 7.6% by Mihret and Samuel 19], in Dangila it was 11.5% by Alemayehu and Mulat [20] and in and around Bahir Dar it was 26.3% by Samuel et al. [21] were evident by coproscopic examination. The report from Bahir Dar Abattoir by Hailu [22] revealed that prevalence 48%; 30.3% by Yalelet [15] and 28.14 by Almaz [23] 28.14% and in Jimma 13.46% prevalence by Abebe et al. [24].

Even if different research findings reported from different parts of the country, considering persistent water bodies of the current study areas, research has not been conducted previously. Therefore, considering the economic importance of the disease, agro ecological variation, different animal husbandry practices and disease prevention and control strategy of the five districts, the current study was designed to determine the prevalence of bovine schistosomiasis (*S. bovis*), to identify potential risk factors for the occurrence bovine schistosomiasis and to characterize pathological lesions induced by *Schistosoma bovis* infection.

**Materials and Methods**

## Study Area Description

The study was conducted in some selected districts of South Wollo Zone (Kalu, Tehuledere and Ambasel Districts) around Ardibo, Logo and Golbo lakes and Borkena river) and Oromia Zone at Cheffa valley. Those study areas were selected purposively based the availabilities of permanent water bodies.

Kalu District is one the administrative districts in south Wollo Zone, Amhara Regional State. The district’s administrative office is found in Kombolcha town which is situated at a distance of 376 km from north of Addis Ababa, 505 km from the Regional capital city, Bahir Dar Town. The district consists of 34 kebeles (30 rural and 4 urban Kebeles). The elevation ranges from 1400 to 1850 meters above sea level. There is bimodal raining pattern in the district and the mean annual rainfall ranges from 700 to 900 mm. The relative humidity of the area varies from 23.9% to 79% and the average minimum and maximum daily temperatures are 11.7ºC and 23.9ºC, respectively [25].

Tehuledere District is among the districts of South Wollo Zone which is located in north eastern Ethiopia. The district is found 431 Km away from the capital of Ethiopia, Addis Ababa and 540 km east of Bahir Dar Town, regional capital city. is The disdrict located at 11º17'N latitude and 39º 42'E longitude, and the elevation of the district ranges from 500 to 2700 meters above sea level with 1200 mm annual rainfall [26].

Ambasel District is one of the 24 administrative districts in south Wollo Zone, Amhara Regional State which is located at a distance of 420 km from Bahir Dar, the regional capital city and 460 Km North of Addis Ababa, capital city of Ethiopia. The district is located at 11º31'05'' North latitude and 39º36'34'' East longitude with an elevation ranges from 1200 to 3200 meters above sea level. The average temperature of the district is 19 ºc and mean annual rain fall (RF) is 500-800 mm which rains in bimodal pattern [27].

Oromia Special zone is found in Amhara Regional State, north eastern Ethiopia. It is located at 325 km North of Addis Ababa, the capital city of Ethiopia. Currently, seven districts are found in this special zone including Dawa Chefa, Bati, Jile Timuga, Artuma Fursi, Dawa Harewa, Bati and Kemise towns. The geographical location of this zone is between 10º01' N to 11°25'N and 39° 41'E to 40°24'E with elevation ranges from 1000 to 2500 meters above the sea level. The mean annual rainfall of the area ranges from 600 mm to 900 mm, and the minimum and maximum temperatures are 12°C and 33°C, respectively [28]. Map of the study sites indicated in Fig 1.

## Study Population

The study animals were cattle in the field and also the cattle that presented for slaughtering in Dessie and Kombolcha municipal abattoirs from purposely selected study sites without discrimination of their sex, age, origin, breed and body condition. The age, sex, body condition, breed and origin of the study animals were recorded as explanatory variables. The age of the individual animal was determined based on their dentition and categorized into three groups: young (<1year), adult (2–5 years) and old (> 5 years) [29]. Breeds were distinguished by their uniquely observable characteristics as described by Bosso *et al.* [30]. Body condition score were recorded as poor, medium and good [31].

## Study design

A cross sectional study was conducted from November 2020 to June 2021 to determine the prevalence of bovine schistosomiasis and associated risk factors.

## Sample size determination and sampling techniques

The total numbers of cattle required for this study were calculated based on the formula given by Thrusfield [32] since there was no recent and enough information on bovine schistosomiasis in the study areas; the sample size required for this study was calculated considering 50% expected prevalence with 5% desired level of precision and 95% of confidence interval with the following formula.

n=1.96^2^ *P_exp_ (1-P_exp_) /d^2^

Where n= Sample size P_exp_= expected prevalence, d= desired absolute precision. Therefore; calculated sample size was 384.

To increase the precision the total number of animals considered for this study were 768 cattle both from the field and the abattoirs. In this study mixed sampling techniques were employed to select the sampled animals. Sampled animals in the field were selected using simple random sampling technique whereas cattle presented for slaughtering from the specified study areas were selected purposively and listed and then systematic random sampling technique with an interval of five was used. The cattle population in each study district was obtained from local government veterinary records and the proportion of cattle from each district were weighted according to the estimated population size in that particular district.

## Data collection and processing

### Coprological examination

Both in the field and abattoirs the study animals were restrained properly and approximately 10 grams of fresh faecal sample was collected directly from the rectum using gloved hands and then the collected fecal samples were transported with cold chain to Wollo University, School of Veterinary Medicine, Parasitology laboratory.

The samples were concentrated using standard sedimentation technique described by Gupta and Singla [33]. 3 g of faeces were taken in to centrifuge tube and 42 ml of water was added and then mixed thoroughly. The suspension was filtered through mish strainer into other centrifuge tube and was left for 15 min. Thereafter, the supernatant was decanted and the sediment was re-suspended. Finally, the sediment was transferred with a pipette to a clean slide and observed under low power (10x) microscope.

### Abattoir survey

Abattoir survey on *S.bovis infection* in 384 cattle was carried out at Kombolcha and Dessie municipal abattoirs, where cattle came from various livestock markets from the selected districts for slaughtering were subjected to coprological examination for the presence and absence of schistosoma eggs and then during post-mortem examination all animals were diagnosed for the presence and absence of adult schistosomes in their mesenteric veins [34]**.** The adult were morphologically characterized using stereomicroscope.

Grossly lesions were identified by visualization, palpation and incision during necropsy. The gross lesions were noted in liver and intestinal tracts and changes in affected organs were conducted depending on their size, color, consistency and shape, as well as other gross abnormalities and representative pieces of tissue from both organs were taken for histopathological examination, however, histopathology was not carried out due to COVID-19 pandemic and other unavoidable difficulties.

# Ethical considerations

An official letter of cooperation was obtained from Wollo University ethical clearance committee. The purpose of the investigation was explained to all eligible animal owners and informed consent was sought from all eligible animal owners to allow their animals for investigation before any procedure is performed. During specimen collection both in the field and the abattoirs all applicable international, national, and/or institutional guidelines for the care and use of animals were followed.

**Data management and analysis**

All the data were entered and coded to Microsoft excel spread sheet and statistical analysis was carried out using SPSS version 20. A descriptive statistic was used to determine the prevalence of the disease in the study area. Chi-square test was computed to see the association of the risk factors with the occurrence of the disease. The test agreement between coprological and post-mortem examination was analysed using Kappa statistics. For all statistical analysis, a p value < 0.05 was taken as significant.

# Result

## Coprological examination results

### Overall prevalence of *S. bovis* infection

Out of the total 768 faecal samples examined (384 from the field and 384 from the abattoirs) 128 (16.7 %) samples were found to be positive for schistosoma eggs and morphologically the eggs were oval to spindle shaped with centrally bulged and terminal spine (Fig 2).

### Association of sex, age, breed and body condition of the Study Animals with *Schistosoma bovis* infection

The prevalence of *schistosoma bovis* infection in the male and female cattle was 15.9% and 18.8% respectively. There was slightly higher prevalence of *schistosoma bovis* in female animals, however; the difference was not statistically significant (P =0.356) as compared with the male animals (**Table 1**). Regarding the age of the study animals, even if slightly higher prevalence (18.4%) was recorded in adult cattle, the present findings showed that the occurrence of *Schistosoma bovis* infection among the three age groups was not statistically significantly differ (P =0.194) (**Table 1**). From this study, it was found that the local breed had high prevalence of 19.0% than the crossbreed which accounts 8.3% prevalence rate, therefore, the recorded prevalence in local breed animals was statistically significantly higher (P= 0.001) than the cross breed animals (**Table 1**). In relation to the body condition of the study animals the current findings showed that the prevalence of *Schistosoma bovis* infection in poor body conditioned animals was found significantly higher (P= 0.000) than in animals with medium and good body conditioned (**Table 1**).

Table 1: Prevalence of Schistosoma bovis Infection with potential risk factors (sex, age, breed and body condition) of the study animals using coprological examination

| **Risk factors** | **No of examined** | **No. of cattle infected** | **(Prevalence (%)** | **χ^2^** | **P-Value** |
| --- | --- | --- | --- | --- | --- |
| **Sex** |  |  |  |  |  |
| **Male** | 571 | 91 | 15.9 | 0.853 | 0.356 |
| **Female** | 197 | 37 | 18.8 |  |  |
| **Age** |  |  |  |  |  |
| **Young** | 104 | 12 | 11.5 | 3.282 | 0.194 |
| **Adult** | 485 | 8 9 | 18.4 |  |  |
| **Old** | 179 | 27 | 15.1 |  |  |
| **Breed** |  |  |  |  |  |
| **Local** | 599 | 114 | 19.0 | 10.963 | 0.001 |
| **Cross** | 169 | 14 | 8.3 |  |  |
| **BCS** |  |  |  |  |  |
| **Poor** | 162 | 47 | 29.0 |  |  |
| **Medium** | 276 | 48 | 17.4 | 28.442 | 0.000 |
| **Good** | 330 | 33 | 10.0 |  |  |
| **Total** | **768** | **128** | **16.7** |  |  |

### Association of Districts/Origins with *Schistosoma Bovis* Infection

In the current study the highest prevalence of *schistosoma bovis* was observed in Dawa Cheffa district (20.6%) and the lowest in Ambasel district (5.2%). The occurrence of schistosoma bovis was found significant (P = 0.046) among the different districts/origins (**Table 2**).

**Table 2: Distribution and Prevalence of Schistosoma bois Infection in the study Districts**

| **Districts** | **No. of cattle examined** | **No. of cattle infected** | **Prevalence (%)** | **χ^2^** | **P-value** |
| --- | --- | --- | --- | --- | --- |
| **Dawa Chefa** | 252 | 52 | 20.6 | 9.699 | 0.046 |
| **Artuma Furci** | 137 | 25 | 18.2 |  |  |
| **Tehuledere** | 221 | 35 | 15.8 |  |  |
| **Kallu** | 100 | 13 | 13.0 |  |  |
| **Ambasel** | 58 | 3 | 5.2 |  |  |
| **Total** | **768** | **128** | **16.7** |  |  |

## Abattoir survey

## Adult worm recovery and comparison between coprological and postmortem examination

During Ante mortem examination, a total of 384 cattle were prescreened for the presence and absence of schistosome parasite eggs using coprological examination and 55(14.3%) cattle were positive **(Table 3**). Of the 384 slaughtered cattle 66 (17.2%) were found to be positive for adult worm in their mesenteric veins during necropsy, and then the adult worms were characterized using stereomicroscopy (**Fig. 3** and **Table 3**). The findings obtained from the current work showed that there was strong measure of agreement (**Kappa (k) =0.775)** between Coprological and post-mortem examination (**Table 3**)

**Table 3:** Abattoir Survey Result (Adult Worm Recovery)

| No of Animals Examined | Coprology Examination | | Post-mortem examination | |
| --- | --- | --- | --- | --- |
|  | No. Positive Animals | Prevalence (%) | No. positive Cattle for Adult Worms | Prevalence (%) |
| 384 | 55 | 14.3 | 66 | 17.2 |
| **Measure of Agreement Kappa=0.775** | | | | |

### Gross pathological findings

In the present study, schistosoma lesions were found in the liver and intestinal tracts.

**Liver:** Most of the pathological findings were tiny whitish foci, which were scattered under the capsule and on the cut surface of the liver. Fibrosis and thickening of the portal tracts were observed in heavily infected animals, the liver was hardened and extensively fibrosed, showing multiple, elevated greyish nodules which consisted of greatly dilated portal veins occupied by hemorrhagic thrombi (**Fig. 4 A and B**)

**The intestinal tracts**: particularly the small intestine, presented a thickened, slightly hyperemic mucosa, with focal petechial hemorrhages and occasional ulcerations. In a few heavily infected cattle, the mucosa was intensely congested and coated with mucous exudate. In some animals superficial intestinal veins were occluded by thrombi, but more often, the vessels were engorged with blood, tortuous and thick-walled (**Fig. 4 C and D**)

**DISCUSSION**

*S. bovis* infection is responsible for considerable economic losses in livestock industry, mainly through mortality, reduced fertility, productivity and stunted growth. The present survey on bovine schistosomiasis covered a considerable area of South Wollo and Oromia special zones. The results based on coprological studies both from the field and abattoirs showed that an overall prevalence of 16.7% (128/768), and based on postmortem examination 17.2% (66/384) slaughtered cattle were positive for schistosome adult worms in their mesenteric veins.

The findings in the present study are in agreement with previous reports of Getachew et *al.* [35] in Kemissie and Yalelet [15] in Bahirdar who reported prevalence of 17.2% and 17.4% respectively. The results obtained both by coprological and postmortem examination are higher when compared with previous findings of Abebe *et al.* [24] 4.49% in Agaro; Almaze [23] 10.93% in Bahir Dar; Zelalem [36] 12.5%, Mengistu *et al.* [18] 10.17%, Mersha *et al.* [16] 13.70% in Fogera; and Mihret and Samuel [19] 7.6% in Debre Tabor. The probable reasons for the higher prevalence in the present study might be due to the presence of known water bodies (lakes) in the selected sites may favor the development and multiplication of intermediate hosts and practicing of free grazing on the pasture land might be another predisposing factor. In support of this, Urquhart *et al.* [37] has explained the importance of water bodies and marshy areas for the occurrence of schistosomiasis.

In the other hand the present findings is lower than the previous studies conducted in Bahir Dar by Amen *et al.* [17] 28%; in Bahir Dar; Almaz and Solomon [38] 37.7% and in Dembia by Alemseged [39] 27.13%. The difference might be due to variation in the accessibility of animals to the marshy area, animal management system, difference in sample size, difference in sampling seasons and difference in the study districts (this might be associated with the difference in their agro-ecology). This is also supported by the explanations of Jesus *et al.* [40] and Narcis *et al.* [41] that the prevalence and occurrence of schistosomiasis in a given area could be influenced by local climate conditions, presence of water reservoirs, lakes, rivers, and availability of suitable final and intermediate hosts.

The association of potential host and environmental factors with the occurrence of S. *bovis* in the selected study sites were assessed and identified. In the present research work prevalence of *S. bovis* was statistically significantly higher (P<0.005) in local breed animals than the cross breed animals. This finding was found to be in line with the previous prevalence studies of Belayneh and Tadesse [42] in Bahir Dar Town; Samrawit [43] in Bahir Dar and its surrounding areas and Alemayehu and Mulat [20] in Dangila district. This might be due to the fact that local breed cattle were kept in outdoor and repeatedly exposed for Schistosoma infection than the crossbred animals, because crossbreds are kept for the purpose of milking in semi-intensive or intensive management systems with the provision of quality concentrate and roughages feed as well as clean water.

In the current study *S. bovis* infection rate showed statistically significant variation (P<0.005) among the three body condition categories. Higher prevalence rate was recorded in poor body condition animals as compared to medium and good body condition animals. This finding in agreement with the previous results reported by Fromsa *et al.* [6]); Abebe *et al.* [24]; Lulie and Guadu [44]; Merawe *et al.* [45]; Belayneh and Tadesse [42]); Mihiret and Samuel [19] and Miressa and Feyissa [46] who confirmed that the infection rate increased with animals which had a poor body condition score. This could be that the acquired immunity status of poor body condition and weak animals becomes more suppressed and susceptible, which might be due to malnutrition and other concurrent infections. Moreover, S. bovis infection can result in weight gain loss and weak acquired immunity [47].

The current research results showed that in the five purposively selected study districts the occurrence and distribution of schistosoma bovis was statistically significantly differ (P<0.005) among the different districts of studied animals. This might be related with the number of studied animals in each district, variation in their animal husbandry practices and variation in their agro-ecology.

In the present study, the sex-wise distribution of *S. bovis* infection did not show any statistical significant variation (P>0.005). This is in agreement with reports in Ethiopia and elsewhere in the world [38, 45, 47, 48]. This might be due to the fact that both sex groups are allowed to graze and drink in similar pasture land and water points, exposing them to the infection comparably. Therefore, the infection appeared to be well distributed between both sex groups.

In the other hand, the current research findings showed that age was not significantly related (P>0.005) to the occurrence of *S. bovis* infection in the studied animals. This is inconsistent with reports of many researchers in Ethiopia and across the world [6, 18, 45, 48- 50] who stated a significant effect of age on *S. bovis* infection in the studied animals and higher prevalence was recorded in old aged animals followed by adult. This could be attributed to the fact that adult and old cattle groups cover large areas and have high grazing capacity than young age groups under extensive and semi intensive management system, where the prevalence of cercariae infection is predominated. However; in the current study as the researchers observed during data collection from the field all age groups were graze in the pasture land, this may leads them for comparable exposure to the infection.

As explained above, in this piece of research work in addition to parasite egg identification adult worms of *S.bovis* were recovered from mesentery veins and the pathology of *S. bovis* was also studied during postmortem examination. Schistosome lesions were characterized in the liver and intestinal tracts. The current findings in agreements with previously reported results of Hussein *et al.* [51] and Almaz *et al.* [52], however; in the present study the recovery of adult worms of *S.bovis* only limited on mesenteric veins rather than considering portal and urinary bladder veins. Generally, the prevalence of bovine schistosomiasis recorded in this study based on coprological and postmortem examination revealed that *S.bovis* is one of the endemic diseases contributing to loss in productivity and production of cattle in the study areas that need serious attention in the future.

# Conclusions

The present study demonstrates that the occurrence of *S. bovis* infection with the overall prevalence of 16.7 % and 17.2 % using coprological and postmortem examination respectively. The infection rate was statistically significantly associated with the body condition score, breeds and origins of the studied animals, but not with the sex and age of sampled animals. Therefore *S.bovis* infection should be taken into consideration as one of the limiting factor for livestock productivity in the study districts. Based on the above conclusions the following recommendations are forwarded for the control of schistosomiasis in cattle and to prevent economic loss.

- Well-planned deworming activities should be practiced in order to reduce the exposure rate of animals to Schistosoma and the impact of schistosome infection in the study districts.
- Direct killing of intermediate host snails with chemicals or destroying their habitats through drainage system should be implemented.
- Awareness should be created for the owners of the animals about the risk factors of the disease and its transmission, as much as possible do not allow grazing their cattle freely on swampy or marsh areas.
- Researches should be encouraged towards the development of vaccines for Schistosoma infection which is considered as control measurement of the disease.

**Author’s contributions**

| No | Name of Authors | Authors contributions |
| --- | --- | --- |
| 1 | Gashaw Molla | Conceived and designed research (developed the proposal, involved in data collection process, conducted the laboratory and wrote the first draft of manuscript |
| 2 | Tarekegn Tintagu | conceived and designed research (developed the proposal), involved in data collection process, conducted the laboratory and wrote the manuscript |
| 3 | Bethelehem Alemu | Edited the manuscript |
| 4 | Ahmed Yasin | Edited the research proposal and manuscript |
| 5 | Alula Alemayehu Assen | Edited the research proposal and manuscript |
| 6 | Kassahun Tadesse | Involved in data collection process |

**Acknowledgments**

The Authors are thankful to Kombolcha regional laboratory administrator for allowing us to conduct the laboratory work and for providing information. We also appreciate and thank district and abattoir administrators of the study sites for their unreserved support during the data collection process.

**Data Availability Statement**

Here in Wollo University there is no data repository system to track online, however; when the data generated and/or analysed during the current study is need the editorial manager or any concerned bodies may get from the corresponding author through email.

**REFERENCES**

1. Solomon A. Prevalence Bovine schistosomosis in and around BahirDar.DVM thesis FVM. Mekelle University, Mekelle Ethiopia. 2008.
2. Tilahun H, and Schmidt E. Spatial Analysis of Livestock Production Patterns in Ethiopia. ESSP II Working Paper 44. Addis Ababa, Ethiopia: International Food Policy Research Institute/Ethiopia Strategy Support Program II. 2012.
3. Jabbar M, Negassa A, Gidyelew T. Geographic distribution of cattle and shoats populations and their market supply sheds in Ethiopia. Kenya: ILRI, Nairobi. Discussion Paper No. 2. Improving Market Opportunities. 2007; p54.
4. Negassa A, Rashid S, Gebremedhin B. Livestock production and marketing in Ethiopia. Ethiopian Support Strategy Program II (ESSP II) Working Paper 26. Washington, D.C. IFPRI. 2011.
5. Juyal PD, Singal LD. Herbal immunomodulatory and therapeutic approaches to control parasitic infection in livestock. Department of veterinary parasitology college of veterinary science Punjab agricultural university Ludhiana-1441004-india. 2011; 1-8.
6. Fromsa A, Behablom H, Meharenet T, Mekibib B. major trematode infections of cattle slaughtered at Jimma municipality abattoir and the occurrence of the intermediate hosts in selected water bodies of the zone. *Journal of Animal and Veterinary Advances.* 2011; **10**(12): 1592-1597.
7. Jejaw A, Zemene E, Alemu Y, Mengistie Z. High prevalence of Schistosoma mansoni and other intestinal parasites among elementary school children in Southwest Ethiopia: a cross-sectional study. *BMC Public Health.* 2015; **15**, 600.
8. Georgs R. Parasitology for veterinarian, eighth edition, ELSEVIER, USA. 2003; 185-200.
9. Taylor MA, Coop RL, Wall RL. Veterinary Parasitology. Third addition. United Kingdom. 2007; 91-94.
10. Okpala HO, Agwu E, Agba MI, Chimezie OR, Nwobu GO, Ohihoin AA. Survey of the Prevalence of schistosomiasis among pupils in Apata and Laranto areas in Jos, plateau state. Online *J Health Allied Scs*. 2004; **1**:1–4.
11. Lefevre CP, Blancou J, Chermette R, Uilenberg G. Infectious disease of livestock. 1^st^ ed. Paris: Lavoisier. 2010.
12. Stothard JR, Stanton MC, Bustinduy AL, Sousa-Figueiredo JC, Van Dam GJ, Betson M, et al Diagnostics for schistosomiasis in Africa and Arabia: a review of present options in control andfuture needs for elimination. Parasitology. 2014; **141**:1947–1961.
13. Tchuem- Tchuenté LA. Control of soil-transmitted helminths in sub-Saharan Africa: diagnosis, drug efficacy concerns and challenges. *Acta Trop.* 2011; **120**:4 –11.
14. World Health Organization. Prevention and control of schistosomiasis and soil-transmitted helminthiasis, WHO technical report series, Geneva. 2002.
15. Yalelet W. Survey on Bovine schistosomosis in and around Bahir Dar, North western Ethiopia. DVM Thesis, faculty of veterinary medicine, Addis Ababa, Debre Zeit, Ethiopia. 2004.
16. Mersha C, Belay D, Tewodros F. Prevalence of Cattle schistosomiasis and Associated Risk Factors in Fogera Cattle, South Gondar Zone and Amhara National Regional State, Ethiopia. *Journal of Advanced Veterinary Research*. 2012; **2**: 53-56.
17. Ameni G, Krok B, Bogale T. Preliminary study on the major bovine trematode infection around Kemissie, Northeastern Ethiopia and treatment trial with praziquantel. *Bull. Anim. Hlth. Prod. Afr.* 2001; 49: 62-67
18. Mengistu S, Tewodros F, Mersha C. Prevalence of Bovine Schistosomiasis in Fogera District, South Gondar Zone, Amhara National Regional State, Northwest Ethiopia. *Glob. Vet.* 2012; **9**(5): 612-616.
19. Mihret T, Samuel D. (Prevalence of Bovine Schistosomiasis and its Associated Risk Factor in and Around Debre Tabor Town, North West of Ethiopia*. E. J. Bio. Scien.* 2015; **7**(3): 108-113.
20. Alemayehu A, Mulat A. Cross-sectional Study on Prevalence of Bovine Schistosomiasis and its Associated Risk Factors in Dangila District, Amhara National Regional State, Ethiopia. *J. Res*. 2015; **5** (3): 397-402.
21. Samuel A, Sefinew A, Samuel D, Yitayew D, Dagmawi YA. Cross Sectional Study on the Prevalence and Possible Risk Factors of Bovine Schistosomiasis in and Around Bahir Dar Town, Northwest Ethiopia. *Europ.J Bio.Scien*. 2016; **8** (1):14-20.
22. Hailu M. Observations on the prevalence and intensity of Schistosoma bovis infection in Bahir Dar area, north central Ethiopia., Addis Ababa University, Faculty of Veterinary Medicine, Debre-Zeit, Ethiopia. DVM Thesis. 1999.
23. Almaz H. Pathology of naturally occurring Schistosoma infection in cattle slaughtered at Bahir Dar municipal abattoir, Northwest Ethiopia., Faculty of Veterinary Medicine, Addis Ababa University, Deber-Zeit, Ethiopia. MSc Thesis. 2007.
24. Abebe F, Behablom M, Berhanu M. [Major Trematode Infections of Cattle Slaughtered at Jimma Municipality Abattoir and the Occurrence of the Intermediate Hosts in Selected Water Bodies of the Zone. *J Ani Vet Advan.* 2011; **10**: 1592-1597.](https://scholar.google.co.in/scholar?cluster=10984851839663288531&hl=en&as_sdt=2005&sciodt=0,5)
25. Yimam DA. Adoption of Forage Innovation in Smallholder Farming Systems: A Case Study in Kalu Woreda of South Wollo Zone, Ethiopia. A Thesis Submitted to Institute of Regional and Local Development Studies of Addis Ababa University in Partial Fulfillment of the Requirement for the Degree of Master of Arts in Regional and Local Development Studies. 2008; 1-137.
26. Adera W, Noah P. Evaluating smallholder farmers’ preferences for climate smart agricultural practices in Tehuledere District, northeastern Ethiopia. *Singapore Journal of Tropical Geography.* 2018; 1-172
27. Melkamu S, Asrat M. Study on the Prevalence of Ovine Fasciolosis in AmbaselWoreda, South Wollo Zone, Amhara Regional State, Ethiopia. *Journal of Animal Research*. 2015; **5**(3): 437-441.
28. Yasine A, Kumsa B, Hailu Y, Ayana D. Mites of sheep and goats in Oromia Zone of Amhara Region, North Eastern Ethiopia: species, prevalence and farmers awareness. *BMC Veterinary Research.* 2015; **11**(122): 1-6.
29. Pope A, George W. Determining the age of cattle by their teeth. Farmers’ Bulleitin 1066, USA Department of Agriculture. 2008; 1-12.
30. Bosso NA, Ciss´e MF, van der Waaij EH, Falla A, van Arendonk, JAM. Genetic and phenotypic parameters of body weight in West African dwarf goat and Djallonké sheep. *Small Ruminant Research.* 2005; **67**.271–278.
31. Nicholson MJ, Butterworth MH. A Guide to Body Condition Scoring in Zebu Cattle. International Livestock Center for Africa (ILCA). Addis Ababa, Ethiopia. 1986.
32. Thrusfield M. Survey in veterinary epidemiology. 2nd ed. Cambridge, Blackwell science Ltd. 2007; 182-189.
33. Gupta SK, Singla LD. Diagnostic trends in parasitic diseases of animals. In: Veterinary Diagnostics: Current Trends. 2012.
34. Urquhart GM, Armour J, Duncan JL, Dunn AM, Jennings FW. Veterinary Parasitology. 2nd ed. *Scotland: Black well science.* 2003; **277**: 117-120.
35. Getachew T, Tesfu K, Berhanu E, Legesse W, Ahmed A, Nega B. Pilot control of fasciolosis and related animal fluke infections by the use of Endod and reduced morbidity: I pre-intervention studies. *Ethiop.vet. J*. 2006; **10**(1):67-70.
36. Zelalem A. Prevalence of bovine schistosomiasis in Fogera Woreda. Business paper submitted to Faculty of Veterinary Medicine, University of Gondar. 2010.
37. Urquhart GM, Armour J, Duncan JL, Jenning FW. Veterinary helminthology veterinary parasitology New York Churchill Livingstone Inc. 1996; 117-119.
38. Almaz H, Solomon W. Repeated simple sedimentation and prevalence of bovine schistosomiasis in selected area of Bahir Dar area. *Ethiopian Veterinary Journal*. 2011; **15**(1): 49 57.
39. Alemseged G, Mebratu A, Tewodros A. The Prevalence of Bovine Schistosomiasis in Dembia Woreda, Ethiopia. University of Gondar, Faculty of Veterinary Medicine, Gondar, Ethiopia.*Act. Parasit.Glob*. 2015; **6**(2): 112-116.
40. Jesus S, Carneiro K, Carneiro C, Brillet P. Evolution of the prevalence of Mansonia Schistosomiasis in the district of Sao Luis doMaranhao (Brazil). *Sante*. 2004; **14**(3): 149-152.
41. Narcis BK, Simon B, Edridah MT. Epidemiology and geography of *Schistosoma mansoni* in Uganda: implication for planning control. *Trop Med Inter Hlth.* 2004; **9**(3):372.
42. Belayneh L, Tadesse G. Bovine schistosomiasis: A Threat in Public Health perspective in Bahir Dar Town, Northwest Ethiopia. *Acta Parasitological Globalis*. 2014; **5** (1): 1-6.
43. Samrawit M. Study on Prevalence and Associated Risk Factors of Bovine and Human Schistosomiasis in Bahir Dar and its Surrounding Areas. *J Ani Res.* 2014; **6**: 967-975.
44. Lulie B, Guadu T. Bovine Schistosomiasis: A Threat in Public Health Perspective in Bahir Dar Town, Northwest Ethiopia. *Acta Parasitologica Globalis.* 2014; **5**: 01-06
45. Merawe M, Amsslu K, Hagos Y, Afera B. Intestinal schistosomiasis of Bovine and Ovine in Fogera district, South Gondar Zone, Amhara National Regional State, Ethiopia. *Act. Parasit.Glob*. 2014; **5**(2): 87-9.
46. Miressa BS, Feyissa BD.  [Prevalence and Associated Risk Factors of Bovine Schistosomiasis in and Around Bakko Town, west Shoa Zone, Oromia, Ethiopia. *Global J Science* F*rontier Research.* 2017; **17**: 58-67.](http://wvj.science-line.com/attachments/article/45/WVJ 7(1) 01-04, Mar 25, 2017.pdf)
47. Niaz S, Tanveer A, Qureshi A. Prevalence of schistosomiasis in cows and buffaloes at different sites of Punjab, Pakistan and its relation to temperature, relative humidity, rainfall and Pan Evaporation. Pak *J Sci.* 2010; **62**: 242-249.
48. Kamanja I, Githigia S, Muchemi G. A survey of *Schistosoma bovis* in cattle in Kwale District, Kenya. *Bull Anim Hlth Prod Afr.* 2011; **59**: 161-168.
49. Pfukenyi D, Mukaratirwa S, Willingham A. Epidemiological studies of *Schistosomamattheei*infections in cattle in the Highveld and Lowveld, communal grazing areas of Zimbabwe. *Onderst J Vet Res.* 2006; **73**(3): 179-191.
50. Islam M, Begum N, Alam M. Epidemiology of intestinal schistosomiasis in ruminants of Bangladesh. *J Bangl Agri Uni,* 2011; **9**(2): 221-228.
51. Hussein MF, Tartour G, Imbabi SE, Ali KE. The pathology of naturally-occurring bovine schistosomiasis in the Sudan, *Annals of Tropical Medicine & Parasitology*. 1975; **69**(2), 217-225.
52. Almaz H, Tamiru N, Asegedech S. Pathology of Natural Infections of *Schistosoma bovis* in Cattle in Ethiopia, Bahir Dar, Ethiopia. *Glob Vet.* 2013; 11 (2): 243-247.
