# Supplementary material for: Bovine schistosomiasis in some selected areas of South Wollo and Oromia Zones of Amhara Region, North-East Ethiopia
Source: PLoS One. 2022 Jun 17;17(6):e0259787. doi: 10.1371/journal.pone.0259787 (PMC9205502; doi:10.1371/journal.pone.0259787)
Supplement: Attachment — Submitted filename: Comments_PONE-D-21-33189.docx [file pone.0259787.s006.docx]

Comments to the Author

1. The study presents the results of original research.

- Yes.

2. Results reported have not been published elsewhere.

- Yes

3. Experiments, statistics, and other analyses are performed to a high technical standard and are described in sufficient detail.

- Moderately addressed

4. Conclusions are presented in an appropriate fashion and are supported by the data.

- Yes

5. The article is presented in an intelligible fashion and is written in standard English.

- Yes, however, there have scope for improvements.

6. The research meets all applicable standards for the ethics of experimentation and research integrity.

- Yes

7. The article adheres to appropriate reporting guidelines and community standards for data availability.

- Yes

Review Comments to the Author

In this study, the authors highlight *Schistosoma bovis* in cattle relating to its the prevalence and to characterize pathological lesions induced by *Schistosoma bovis* including risk factor assessment for the animal-level infestation of South Wollo and Oromia Administrative Zones, Amhara regional state, Ethiopia via a cross sectional survey. The study shows the occurrence of *S. bovis* infection with the overall prevalence of 16.7 % and 17.2 % using fecal sample examination and postmortem examination, respectively. The infection rate was statistically significantly associated with the body condition score, breeds and origins of the studied animals, but not with the sex and age of sampled animal. The overall outlook of the study is interesting. However, there are some lacunae in this manuscript that are needed to address before publication of the manuscript.

Structure of the manuscript:

- The authors did not fully follow journal guidelines for formulation of manuscripts like no line number, reference style including Table and Figure captions.
- The title of the study may be “Bovine schistosomiasis in selected areas of south Wollo and Oromia Zones of Amhara Region, North-East Ethiopia”
- Abstract first line *Schistosoma bovis,* scientific name should be in italic
- Number of the words should reduce to ≤ 300 words

Introduction:

- Include some livestock statistics under reference [1 ,2]
- Kindly add few more lines in this paragraph otherwise merge with the third paragraph
- Schistosomiasis is one of the trematode infestation (not infection) (3^rd^ paragraph 1^st^ line). And put a full stop after infestation.
- Kindly paraphrase these lines under the reference no. [15] and [17] to [21]

Materials and Methods

- Avoid vague words like “some selected districts” (confirm the study locations)

Study population

- Kindly include operational definition of poor, medium and good in a supplementary file.

Sample size determination and sampling techniques

- Clear these sentences under this section

Abattoir survey

- Grossly lesions were identified by visualization, palpation and incision during necropsy: include a reference

Ethical considerations

- Kindly incorporate the number and date for ethical approval taken

Data management and analysis

- Cohen’s kappa statistics?

Result

Overall prevalence of *S. bovis* infestation

- Kindly put a table and demonstrated the prevalence rate with 95% Confidence Interval (CI)
- “Association of sex, age, breed and body condition of the Study Animals with Schistosoma bovis infection” shorten the title as “association of risk factors with the animal-level positive status of *Schistosoma bovis*”

Abattoir survey

Adult worm recovery and comparison between coprological and postmortem examination (Table 3)

- This is strong measure of agreement or substantial agreement? The value of kappa (k) ≤ 0 as indicating no agreement and 0.01–0.20 as none to slight, 0.21–0.40 as fair, 0.41– 0.60 as moderate, 0.61–0.80 as substantial, and 0.81–1.00 as almost perfect agreement and with a P value 0.05 was considered as statistically significant(McHugh, 2012).
- Kindly mention p value for the measurement.
- The authors should calculate sensitivity (Se) and specificity (Sp) as well as positive and negative predictive values (PPV and NPV) of two tests (fecal sample examination and postmortem examination) from test outcomes for better presentation of study results.

Conclusions

- Kindly reduce the reductant text in the conclusion section
