# Supplementary material for: Bovine schistosomiasis in some selected areas of South Wollo and Oromia Zones of Amhara Region, North-East Ethiopia
Source: PLoS One. 2022 Jun 17;17(6):e0259787. doi: 10.1371/journal.pone.0259787 (PMC9205502; doi:10.1371/journal.pone.0259787)
Supplement: Attachment — Submitted filename: Response to Reviewers edited .doc [file pone.0259787.s007.doc]

**Rebuttal letter**

**Dear Respected academic editors and reviewers**

Thank you for giving us the opportunity to submit a revised draft of the manuscript “**Bovine Schistosomiasis in Some Selected Areas of South Wollo and Oromia Zones of Amhara Region, North-East Ethiopia**” We appreciate the time and effort that you providing feedback on our manuscript and we are also grateful for the insightful comments and valuable improvements to our paper. Considering both the academic editors and reviewers comments all section of the manuscript are thoroughly revised and mistakes are corrected. Those changes are highlighted with yellow color and track changed in the revised manuscript.

**Response for the academic editor**

**Dear Respected academic Editor**

1. We thank you so much for giving us the chance to revise our manuscript. As you explained that our first submitted manuscript was not fully meet PLOS ONE’s publication criteria, we apologies for the inconvenience, but now we have tried to follow the format.
2. Regarding the text overlap of our submission and the previously published works, we have admitted that but now we have revised and cited our sources as we can as possible.
3. As per your comment the study design and study population under materials and methods has merged (**line no 168-176**)
4. Data analysis: we really thank you so much for your high level professional comments and advise. As per your comments by considering of the district as random intercept the data is analysed using **univariable mixed effect logistic regression** and then variables with a p-value <0.25 in the univariable screening included in the **multivariable mixed effect logistic regression** analysis. We have also described the methods of model selection, model fit, collinearity, confounding and interaction. Data analysis about the abattoir survey using coprological and postmortem methods, we have also evaluated the efficacy/performance of sedimentation technique by calculating it’s sensitivity, specificity, positive predictive value and negative predictive value] considering postmortem as a gold standard test.

**Response for reviewer #1**

**Title**: The comment give on the title is accepted by the authors and the first title “**Study on Bovine Schistosomiasis in Some Selected Areas of South Wollo and Oromia Zones of Amhara Region, North-East Ethiopia**” rephrases to “**Bovine Schistosomiasis in Some Selected Areas of South Wollo and Oromia Zones of Amhara Region, North-East Ethiopia**”, the word “study on” is removed.

**Overall structure of the manuscript:** The comments forwarded by the respected reviewer about the structure of the manuscript are fully accepted and now corrected as per the guidelines of the journal. Throughout the manuscript scientific names are thoroughly checked and italized.

**Abstract:** All the correction measures given by the reviewer are accepted and the authors tried to shorten and modified the whole section of the abstract.

**Introduction**:

- As suggested by the reviewer, we have added and reviewed the livestock statistics/data in the first paragraph of the introduction section (**Line no 51-60**)
- The reviewer kindly requested us to add few more lines in this paragraph “Parasitic diseases have been indicated among the major constraints in animal production throughout the tropical and sub-tropical countries of the world. Trematodes in general and schistosoma in particular have been reported as one of the major problems both in animals and humans around the world [5-7]”. As per the comments of the reviewer we have modified and merged with the paragraph highlighted in green color. The geographical distribution of bovine schistosomiasis has been determined primarily by the distribution of snail intermediate host particularly Bulinus species which are important for the occurrence of disease in bovine species. It has been indicated as endemic in sub-Saharan Africa and also found commonly in northern, eastern, southwestern and central parts of Ethiopia [8-10]. The endemicity of the disease particularly in the area with large permanent water bodies and marsh pasture area which anticipated for being ideal for the propagation and maintenance of the intermediate host (snails) has been found responsible for high prevalence of the infestation [11-12]. Out of 10 species reported to naturally infested cattle, six have received particular attention mainly because of their recognized veterinary significance; Schistosoma bovis, S.indicum, S. japonicum, S. matthei, S. intercalatum,  S. nasale and S. rodhoni . S. bovis, S. matthei and  S. intercalatum are the most important *s*pecies that can cause *s*chistosomiasis in ruminants [7]. (**line no 76-87**)
- As suggested by the reviewer the phrase “**schistosoma infection**” is changed into “schistosoma infestation” not only in the introduction part but also throughout the manuscript
- As per the recommendation of the reviewer the paragraph highlighted in red color “The prevalence of *Schistosoma bovis* has been reported from different regions of the country by a number of authors; 28% in Kemissie by Ameni et al. [17] in Fogera it was 10.17% by Mengistu et al. [18], and 13.70% by Mersha *et al.* [16], in Debre Tabor it was 7.6% by Mihret and Samuel 19], in Dangila it was 11.5% by Alemayehu and Mulat [20] and in and around Bahir Dar it was 26.3% by Samuel et al. [21] were evident by coproscopic examination. The report from Bahir Dar Abattoir by Hailu [22] revealed that prevalence 48%; 30.3% by Yalelet [15] and 28.14 by Almaz [23] 28.14% and in Jimma 13.46% prevalence by Abebe et al. [24]’ Paraphrased into the paragraph highlighted by green color “The prevalence of *Schistosoma bovis* has been reported from different regions of Ethiopia. Accordingly, prevalence of 28% in kemisse [24], 10.17% in Fogera [25], 13.70% in Fogera [12], 7.6% in Debretabor [26], 11.5% in Dangila [20], 26.3 % in Bahir Dar [27], 48% in Bahir Dar [28], 30.3% in Bahir Dar [10], 28.14% in Bahir Dar [29] and 13.46% in Jimma [30] have been reported both by coprological and postmortem examinations” (**line no 118-123 of the manuscript**).

**Materials and methods**

**Study area description:** as per the recommendation of the reviewer 1 to avoid vague words like “**some selected districts**”; authors have tried to correct **the sentence “**The study was conducted in the selected districts of South Wollo Zone such as Kalu, Tehuledere and Ambasel Districts around Ardibo, Logo and Golbo lakes and Borkena river) and Oromia special Zone of Dawa Cheffa and Artuma furci”

**Study population:** the reviewer 1 forwarded comment to put the operational definition of poor, medium and good body condition animal classification as a supplementary file, therefore, we have fully agreed with the reviewer comment and prepared as supplementary file (**S2**)

**Sample size determination and sampling techniques**: as per the suggestion of the reviewer to make clear these sentences under this sub- section, we have revised and rewrite again (**line** **no** **179**-**189**)

**Abattoir survey:** as per the comment of the reviewer 1 to put reference for the gross lesion characterization, we have characterized the gross lesion using a method (visualization, palpation and incision) as described by Di Provvido et al. (2018) (**line no 219 reference 41**)
**Ethical considerations**: the reviewer kindly requested us to incorporate the number and date for ethical approval taken, therefore; we have been received the ethical clearance from Wollo University ethical clearance committee a letter dated on 12, October, 2020 with a reference number of WU/CMHS13/105/2020 (**line no 226**)

**Data management and analysis**: for the measurement of agreement between sedimentation technique and post-mortem examination the reviewer advised us to use Cohen’s kappa statistics, therefore; the authors have accepted the recommendation and analysed using it and the efficacies of the two tests are also calculated. In addition to this as per the recommendation of the academic editors the data is analysed both by univariate and multivariate mixed effect logistic regression analyses. (In the revised version of the manuscript whole section of “**Data management and analysis**” is modified (**line no 233-278**)).

**Result**

As per the recommendation of the editors and reviewers **major modification** has made on the result section.

**Prevalence of *S. bovis* infestation:** the reviewer 1 recommended us to demonstrate the prevalence rate (percentage) with 95% CI, as per the recommendation of the reviewer we have calculated 95% CI for all percentages and presented in **table 1**.

# As suggested by the reviewer 1 the title “Association of sex, age, breed and body condition of the Study Animals with *Schistosoma bovis* infection” is modified and shortens into “Risk factor analysis” (line no 288)

**Abattoir survey:**

- In the comparison between sedimentation technique and postmortem examination as the value of kappa described by McHugh, (2012), the reviewer recommended us to change the strong measure of agreement into substantial agreement and we have corrected it and P-value is calculated (**described from line no 338-345 and table 4**).
- With the same to the academic editor the reviewer 1 recommended us to calculate sensitivity (Se) and specificity (Sp) as well as positive and negative predictive values (PPV and NPV) of two tests (sedimentation technique) and postmortem examination), therefore; for better presentation of study result we have calculated the efficacy of sedimentation technique (Se, Sp, PPV and NPV) by considering postmortem examination method as a gold standard **(described from line no 348- 354 and table 5**).

**Conclusions:**

As per the recommendation of the reviewer we have paraphrased the conclusion section.

**Author’s contributions**: we have followed the journal style.

**Response for reviewer #2**

**General comments**: as per the comments of the reviewer 2 we have thoroughly checked the whole document and italized the scientific names

**Abstract:**

- As the reviewer 2 commented us the description regarding the abattoir survey is too long and difficult to understand and realize the meaning; therefore; by accepting the comments given by the reviewer 2 we the authors have tried to revised and make clear to the readers (**line no 23-25**)
- The reviewer also commented us to provide 95% CI for the overall prevalence and the measure of agreement between coprological and post-mortem examination. The comment forwarded by the reviewer welcomed by the authors and the 95% CI is calculated and included in the manuscript.
- Generally we have made major modification on the abstract section by considering the comments forwarded by the editors and reviewers.

**Introduction:**

- In paragraph one of this sections the same to reviewer 1 the reviewer 2 also recommended us to incorporate the total cattle population. Therefore; we the authors full agreed with the thoughts of the reviewer and included Ethiopian’s livestock population and reviewed again (**line no 51-60**).
- Supported with references we the authors have been explained that livestock sector in Ethiopia is not efficiently and fully exploited due to several constraints like malnutrition, traditional husbandry practice, poor genetic makeup and prevailing diseases. But the reviewer 2 commented us to add few more information about the availability or limitations of the veterinary services to diagnose and treats the animal diseases in Ethiopia. As per the recommendation of the reviewer 2 we have reviewed and included information about availability or limitations of the veterinary services to diagnose and treat the animal diseases (**line no 64-71**).
- In addition the reviewer 2 also commented us to write a paragraph on different clinical signs found at different stage of schistosomiasis in cattle and the comment is well accepted and the clinical signs in the three stages of schistosomiasis reviewed and incorporated in the revised manuscript **(line no 88-101**)

Objectives:

The reviewer 2 commented us the study objectives are not specific and needs modification. Comment is accepted and tried to modify and make them specific including the name of the study sites (**line no 128-132**)

**Materials and Methods:**

**Study area description:** The same to the first reviewer the second reviewer also recommend to avoid vague words like “**some selected districts**”, therefore; authors have tried to correct the sentence as **“**The study was conducted in the selected districts of South Wollo Zone such as Kalu, Tehuledere and Ambasel Districts around Ardibo, Logo and Golbo lakes and Borkena river) and Oromia special Zone of Dawa Cheffa and Artuma furci”

**Study map:** The reviewer 2 commented us on the map (Fig 1) as the Amhara region is looking abnormally bigger than others. Considering the comment forwarded by the reviewer we have checked and remapped, attached in **fig1**.

Study population: The reviewer is forwarded his comment as it is quite difficult to understand the district name of Dessie and Kombolcha municipal abattoirs, where they are situated? We the authors are fully agreed with the reviewer comment and both Dessie and Kombolcha municipal abattoirs are described under the abattoir survey sub-heading (**line no 211-213**).

**Study Design:** Reviewer 2 suggested us objectives should not be included in the study design, therefore; authors accepted the comment given by the reviewer and objectives already removed from this section. But as per the recommendation of the editor s**tudy design and population** are merged as a one sub-heading (**line no 169-176**)

**Data collection and processing**

- As per the recommendation of the reviewer 2 “Data collection and processing” sub-heading is modified into two separate sub-headings which are **sample collection (line no 196)** and **parasite egg detection** (**line no 201**)
- **Coprological method:** The reviewer 2 also recommended us to specify the kinds of coprological methods used for this paper. We sincerely appreciate the valuable comments and suggestions given by the reviewer. We have used the **sedimentation technique** and explained under sub-heading of “parasite egg detection” (**line no 202-208**)

# Ethical considerations:

# In the draft manuscript we have indicated that an official letter of cooperation was obtained from Wollo University ethical clearance committee but under this sub-heading the reviewer 2 suggested us to include the issuing no. of the official letter. Therefore, as the suggestion of the reviewer we have included the issue no of the official letter which is WU/CMHS13/105/2020 (line no 226).

Data analysis: As the test agreement between coprological and post-mortem examination was analysed using Kappa statistics but the reviewer recommended us to mention the different kinds of agreements like weak, moderate, good and strong agreement with references. As per the reviewer recommendation we mentioned that the values ≤ 0 as indicating no agreement and 0.01–0.20 as none to slight, 0.21–0.40 as fair, 0.41– 0.60 as moderate, 0.61–0.80 as substantial, and 0.81–1.00 as almost perfect agreement and with a P value 0.05 was considered as statistically significant (McHugh, 2012) (**line no 274-278).** In addition to this the risk factor identification was analysed using univariate and multivariate mixed effect logistic regression (**line no 235-270**) and efficacy of sedimentation method (Se, Sp, PPV, NPV) were calculated considering postmortem examination as a golden standard (**line no 271-274)**

Result

As per the recommendation of the editors and reviewers authors have made major modification in the result section.

**Prevalence or percentage values of *S. bovis*:** The reviewer suggested us to provide 95% CI for all the prevalence or percentage values of *S. bovis* mentioned throughout the manuscript. We think this is an excellent suggestion. We have calculated 95% CI for all percentage values of *S.bovis* and presented in **table 1**.

**Association of potential risk factors and Schistosoma bovis infestation:** as per the suggestion of both the reviewer 1 and 2 this sub-heading is modified and shorten into “risk factor analysis” (**line no 288**) and risk factors which have statistical significant values (p<0.005) are explained first and followed by the insignificant values (**line no 289-295**)

**Figure 4:** The reviewer recommended us to use indicator to identify the specific lesions in the figures as we did for figure 3. Thank you for pointing this out, we have indicated the specific lesions both in the liver and intestinal tracts (**figure 4**)

**Discussion:**

- In our draft manuscript we have wrongly given the same explanation/justification for both the lower and higher prevalence records when compared to the previously reported findings, which is commented by the reviewer 2. We thank you for insightful comment. Considering the comments forwarded by the reviewer we have carefully seen and provided logical and scientific explanation how the present prevalence records is higher and/or lower in comparison with previously reported findings and cited appropriately, which is marked and track changed in the revised manuscript (**line no 400-404 for higher prevalence and 407-412 for lower prevalence record**)
- In the draft manuscript we have indicated that as the association of potential host and environmental factors with the occurrence of *S.bovis* were assessed and identified. But the reviewer 2 complained that as he did not find any environmental factors that we have explored through this study. However; we have been considered that the origin of the animals as an environmental factor, but we have convinced by the thought of the reviewer and description about the environmental factors is removed from the revised manuscript.
- The reviewer recommended us to put references supporting our opinion regarding the justification that we have given how local breed is stastically significant and crossbreed is not. As per the recommendation of the reviewer we have put supportive reference in the revised manuscript (**line no 420 reference number 61**)

Conclusion and recommendations:

The same to reviewer 1 the reviewer 2 also suggested us to put in a single paragraph without enlisting the recommendations. We are fully agreed with both reviewer and corrected accordingly.
