# Supplementary material for: Bovine schistosomiasis in some selected areas of South Wollo and Oromia Zones of Amhara Region, North-East Ethiopia
Source: PLoS One. 2022 Jun 17;17(6):e0259787. doi: 10.1371/journal.pone.0259787 (PMC9205502; doi:10.1371/journal.pone.0259787)
Supplement: Attachment — Submitted filename: Response to Reviewers.docx [file pone.0259787.s008.docx]

**Academic Editor's comments:**

Lines 23-27:

Please rephrase as suggested below:

An abattoir survey was carried out on 384 cattle to evaluate the performance of the sedimentation method. The risk factors were identified using multivariable mixed-effect logistic regression analyses.

Please report here the overall prevalence of coprological examination results which also includes the abattoir part. Then report the true prevalence of schistosomiasis based on postmortem results.

***Thank you for your suggestions and comments forwarded. The suggested comment were included in the improved manuscript from L24-29.***

Please delete lines 31-41: starting from  "The prevalence" up to "body condition animals"

Please rewrite this part by interpreting the odds ratio of three significant variables [breed, body condition, and age] in the multivariable model.

***Thank you for the suggestion and comment. We have deleted line 31-41 of the manuscript written from L31-34.***

Please conclude based on the risk factors. Like, local cattle with medium and poor body condition should be prioritized for deworming and future surveillance.

***We are grateful for the suggestions and included the suggestion in the improved manuscript from L37-38.***

Please delete lines 233-235 up to the citation of R: As univariable mixed-effect logistic regression analyses were performed to assess the association between the outcome and explanatory variables, the Chi-square test is not needed.

***Thank you for the suggestions and we did the same as per the suggestions in the improved manuscript.***

Line 237 and elsewhere in the manuscript: Please replace univariate with univariable and multivariate with multivariable.

***We replaced univariate with univariable and multivariate with multivariable as per the suggestions in the improved manuscript.***

Please cut lines 243-250 and paste them at the beginning of the data management and analysis.

***Thank you for the suggestion. We cut and paste the stated statement in the improved manuscript from L214-221.***

Please delete lines 273-274: Also, the likelihood ratio (LR) was determined for evaluating the validity of the sedimentation technique using the epiR package.

Please add the likelihood ratio in the previous sentence and cite 48 as all of these parameters were estimated using a function of this package.

***We have deleted the lines and included the suggestions in the improved manuscript from L248-250.***

Please delete lines 274 [starting from the "The test] to 278. It is no longer needed when the performance of the sedimentation technique was evaluated which is more informative just than just testing the agreement.

***Thank you very much for the suggestion and we did the same in the improved manuscript.***

Please delete lines 289-295. Please add here the results of the univariable screening. Just mention the name of variables included in the multivariable model citing Table 1.

***Thank you for the comments and we have included the suggestions in the improved manuscript from L260-265.***

Please merge Tables 1 and 2. It can be done by deleting the last two columns of Table 1 and replacing them with the last two columns of Table 2.

***We have merged the two tables and the merged table was presented in L268-270***

The odds ratio estimate shown in Table 2 for sex and age are incorrect.

For example, the prevalence of schistosomiasis in male cattle is 15.9%. The male category was considered as a reference [1]. However, the prevalence of schistosomiasis in female cattle is 18.8% higher than that of male cattle. So, the odds ratio for female cattle must be >1 but the authors presented <1. This is also the same for age. In addition, it is better to consider the "young" category of age as a reference for having the lowest prevalence.

***Thank you for the comments and suggestions. We have corrected the comment as per the suggestion in the improved manuscript version in L268-270.***

Please delete this sentence from lines 320-321. They have a significant effect (p<0.05) on bovine Schistosoma.

***We have deleted the lines as per the suggestions in the improved manuscript***

Please delete this sentence from lines 324-325:  Furthermore, young cattle had lower odds (OR=0.64, 95% CI, 0.30-1.35) than old cattle to be shistosoma positive. Because the estimation of the odds ratio for the age group was not correct and also the mentioned odds ratio was not significant. Please recalculate and consider young age as a reference.

***Thank you for the suggestions and we have recalculated the odds ratio of the age category in the improved manuscript from L279-281***

Please rename the title of Table 5: Evaluation of the efficacy of sedimentation technique for the diagnosis of bovine schistosomiasis (postmortem examination as reference test)

***We have renamed the title of Table 3 in the improved manuscript***

Please delete this sentence and Table 4: The findings obtained from the current work showed that there was substantial agreement (Kappa = 0.775) between coprological (sedimentation technique) and post-mortem examination.

***We are grateful for the suggestions. We have deleted Table 4 and the lines as per the suggestion in the improved manuscript***

Please delete lines 431-435 as gender was not significantly associated.

***Thank you for the suggestion. We have deleted the lines in the improved manuscript***

Lines 436-445: Here authors made a self-contradictory discussion. Table 3 shows age as a significantly associated variable [although the mentioned odds ratio is not correct] but they are discussing it as non-significant.

***We are sorry for the discrepancies created and we have corrected the concern in the improved manuscript in L375-383***

Please rephrase the long sentence in lines 446-448.

***We have rephrased the sentence in the improved manuscript from L382-384***

Please rephrase the sentence in lines 449-452 [starting from "The current"]

***Thank you for the suggestion and we have corrected it as per the suggestions in L384-386.***

Please delete the lines 453-459 as these are results. Please discuss here the six false-positive coprological examination results.

***We have deleted the lines as per the suggestions and included the suggestions in L386-388***

lines 467-475:

Please rewrite the conclusion based on the results as also suggested earlier.

***The conclusion of the manuscript was written again considering the suggestions and comments earlier in the improved manuscript in L394-407***

**Review Comments to the Author**

Reviewer #1: The manuscript substantially improved. However, typo-editing errors (scientific name, odd ratio, p value where appropriate) present throughout the text and tables. Author(s) should fix this issue before publication. Additionally, authors should check the plagiarism issue in the final version.

***We the authors are indebted for the positive complement to the level of improvement of the manuscript. We have also made editions on the concerns raised.***

Reviewer #2: Thanks to the authors, they have addressed all the inquiries. Now the manuscript is looking better. I have no further inquiries.

***We thank you very much***
