# Supplementary material for: Bovine schistosomiasis in some selected areas of South Wollo and Oromia Zones of Amhara Region, North-East Ethiopia
Source: PLoS One. 2022 Jun 17;17(6):e0259787. doi: 10.1371/journal.pone.0259787 (PMC9205502; doi:10.1371/journal.pone.0259787)
Supplement: Attachment — Submitted filename: Response to Reviewers.docx [file pone.0259787.s009.docx]

**Academic Editor's comments:**

Please rephrase Lines 35-38 as given below:

Local cattle breed (OR= 2.44, 95%CI= 1.34-4.43), poor body condition (OR= 4.09, 95% CI=2.45-6.83) and adult (OR=1.78, 95% CI= 1.21-3.28) cattle are more likely to acquire schistosomiasis than crossbreed, good body condition, and young cattle.

***Thank you for your suggestions and comments forwarded. The suggested comment was included in the improved manuscript from L35-38.***

Similarly, please rephrase lines 41-42:   In conclusion, adult local cattle with medium and poor body conditions should be prioritized for deworming and future surveillance.

**Thank you for the suggestion and incorporated it into the improved manuscript in L41**

Lines 58-63: Please rephrase as, In addition, the existing veterinary service delivery under the current animal health system is considered to be unsatisfactory in both private and public sectors because of the low government attention, inadequate budget allocation, gaps in control of illegal drug circulation, implementation of ethical practices, and shortage of basic laboratory facilities [5].

***Thank you for your suggestions and comments forwarded. The suggested comment was included in the improved manuscript from L58-61.***

Please rephrase lines 77-81: Out of 10 species reported as naturally infesting cattle, six have received particular attention mainly because of their recognized veterinary significance: please mention those six with veterinary significance [7].

**We are grateful for the suggestion and incorporated in the improved manuscript from L… to L76-78.**

Please delete lines: 267-270: "However, the sex of the cattle doesn’t have a significant association (P>0.05) with S. bovis. Accordingly, local breed cattle (OR=2.60; 95% CI= 1.45– 4.67) with poor body condition (OR= 3.70; 95% CI= 1.25–4.18) have a higher risk of acquiring the disease than crossbreed with good body condition cattle."

**Thank you for the comment and we have deleted the statement in the improved manuscript.**

Please start the discussion with the major findings and their importance to control bovine schistosomiasis.

**Thank you for the suggestion and we started the discussion part with major findings in the improved manuscript in L331-335.**

Line 363L Please delete "statistically"

**The mentioned word was deleted from the improved version of the manuscript.**

Please delete "rate" from lines 371, 372, and 376 as prevalence is a proportion [not a rate}.

**We have deleted the word from the improved manuscript version.**

False positivity is related to specificity, not sensitivity. So, if no proper explanation exists then better to delete this sentence: "In this study, six (6) false positive coprological examination results were which might be to the sensitivity (74.24%) of the coprological test relative to postmortem examination."

**We have deleted the statement from the improved manuscript version.**

Please delete lines 400-401 as they are general statements, not conclusions: "This study aims to determine the prevalence and associated risk factors of S. bovis in cattle.  In addition, the pathological lesions caused by S. bovis were characterized. Accordingly,"

**We have deleted the lines as per the suggestion.**

Also, please delete these sentences as they are results, not conclusions: The disease is significantly associated with the age, breed, and body condition of the animals. As a result, adult local cattle with poor body conditions animals were at higher risk to acquire the disease.

**Thank you for the suggestion and we have deleted it from the improved manuscript version.**

Please rephrase as, For effective prevention and control of the disease, adult local cattle with medium and poor body conditions should be prioritized for deworming and future surveillance.

**Thank you for the suggestions and we have rephrased the statement in the improved manuscript version in L394-396.**
